# A Chinese Variant Marek’s Disease Virus Strain with Divergence between Virulence and Vaccine Resistance

**DOI:** 10.3390/v9040071

**Published:** 2017-04-03

**Authors:** Guo-rong Sun, Yan-ping Zhang, Hong-chao Lv, Lin-yi Zhou, Hong-yu Cui, Yu-long Gao, Xiao-le Qi, Yong-qiang Wang, Kai Li, Li Gao, Qing Pan, Xiao-mei Wang, Chang-jun Liu

**Affiliations:** Division of Avian Immunosuppressive Diseases, State Key Laboratory of Veterinary Biotechnology, Harbin Veterinary Research Institute, Chinese Academy of Agricultural Sciences, Harbin 150069, China; sgrshenhua@hotmail.com (G.-r.S.); zhyp_77@hvri.ac.cn (Y.-p.Z.); lv6739533@163.com (H.-c.L.); zlyi123321@126.com (L.-y.Z.); cuihy@hvri.ac.cn (H.-y.C.); ylg@hvri.ac.cn (Y.-l.G.); qxl@hvri.ac.cn (X.-l.Q.); yqw@hvri.ac.cn (Y.-q.W.); likaihvri@163.com (K.L.); gaoli0820@163.com (L.G.); panqing20050101@126.com (Q.P.)

**Keywords:** Marek’s disease virus, vaccination failure, virulence, pathogenicity, vaccine efficacy, evolution

## Abstract

Marek’s disease (MD) virus (MDV) has been evolving continuously, leading to increasing vaccination failure. Here, the MDV field strain BS/15 was isolated from a severely diseased Chinese chicken flock previously vaccinated with CVI988. To explore the causes of vaccination failure, specific-pathogen free (SPF) chickens vaccinated with CVI988 or 814 and unvaccinated controls were challenged with either BS/15 or the reference strain Md5. Both strains induced MD lesions in unvaccinated chickens with similar mortality rates of 85.7% and 80.0% during the experimental period, respectively. However, unvaccinated chickens inoculated with BS/15 exhibited a higher tumor development rate (64.3% vs. 40.0%), but prolonged survival and diminished immune defects compared to Md5-challenged counterparts. These results suggest that BS/15 and Md5 show a similar virulence but manifest with different pathogenic characteristics. Moreover, the protective indices of CVI988 and 814 were 33.3 and 66.7 for BS/15, and 92.9 and 100 for Md5, respectively, indicating that neither vaccine could provide efficient protection against BS/15. Taken together, these data suggest that MD vaccination failure is probably due to the existence of variant MDV strains with known virulence and unexpected vaccine resistance. Our findings should be helpful for understanding the pathogenicity and evolution of MDV strains prevalent in China.

## 1. Introduction

Marek’s disease (MD) is a lymphoproliferative disease in chickens that has caused considerable economic losses to the commercial poultry industry worldwide. The MD virus (MDV) is the etiological agent of MD. It is an oncogenic alpha-herpesvirus that belongs to the genus *Mardivirus* and G*allid herpesviruses* species, which includes G*allid herpesvirus* type 2 (GaHV-2; represented as MDV in this manuscript), GaHV-3, and M*eleagrid herpesvirus* type 1 (MeHV-1; represented as HVT in this manuscript) [1]. However, GaHV-3 and MeHV-1 are non-oncogenic, with only MDV causing the disease in susceptible hosts. Moreover, MDV can be further classified into four pathotypes, including mild (m), virulent (v), very virulent (vv), and very virulent plus (vv+) MDV strains, based on the induction of MD lesions in unvaccinated and vaccinated chickens, in line with the MDV pathotyping assay developed at the Avian Disease and Oncology Laboratory (ADOL) of the United States Department of Agriculture [2,3]. The virulence and vaccine resistance of the MDV strains are generally associated, i.e., the more virulent MDV strains are more likely to induce more serious MD lesions in susceptible chickens, with the associated vaccines less likely to provide protection.

Vaccinations are the primary approach used for controlling MD in chickens, and three types of vaccines have been developed against MD, including HVT, nonpathogenic GaHV-3, and attenuated GaHV-2 [4,5,6]. MD has been successfully controlled by vaccination, significantly reducing economic losses to the domestic poultry industry [7,8]. However, the vaccines used for MD control cannot induce sterile immunity and allow for replication and transmission of virulent MDV strains in a live host, leading to complicated interactions between the pathogens, vaccines, and hosts [9,10,11,12]. Thus, MDV has continued to evolve and obtain enhanced virulence over the last few decades owing to the widespread use of these vaccines. The relationship between increased MDV virulence and the introduction of different vaccines was summarized as a step-wise evolution, in which MDV strains have shown continuous evolution to maintain virulence, acquiring the ability to overcome immune responses induced by vaccines [13]. Successive generations of MD vaccines have been introduced to protect birds from increasingly virulent MDV strains; however, the virus has countered each new vaccine.

In China, MDV was first reported in the 1970s. At present, laying and breeding chickens are vaccinated with vaccine CVI988 (an effective attenuated GaHV-2 vaccine used commercially worldwide), 814 (an effective attenuated GaHV-2 vaccine used widely in China), or a bivalent vaccine (CVI988 plus HVT) via subcutaneous or intramuscular injection at the time of hatching in the vast majority of Chinese poultry enterprises. The economical HVT vaccine is normally used to vaccinate commercial meat-producing chickens, which are typically raised for only 7–8 weeks and have a lower risk for MD. Although the use of vaccines has effectively controlled MDV, MD vaccination failure cases have occasionally occurred, although little is known about the causes of vaccination failure.

In this study, we isolated a field MDV strain BS/15 from severely diseased chickens that were vaccinated with the commercial vaccine CVI988. Furthermore, the pathogenicity of BS/15 to specific pathogen-free (SPF) chickens was analyzed, and the protective efficacy of vaccine CVI988 and vaccine 814 against BS/15 was evaluated. Our study was created in order to increase understanding of the pathogenic characteristics of the newly isolated MDV strain, the causes of MD vaccination failure, and the evolution of MDV, as well as providing guidance for MD control.

## 2. Materials and Methods

### 2.1. Collection of Clinical Samples

A layer flock in Baishan City of the Jilin Province in China developed serious MD in 2015. Chickens had been vaccinated with the commercial vaccine CVI988 at 1 day of age, and clinical symptoms of MD were observed beginning at about 55 days of age. The incidence of MD in the chicken flocks quickly reached about 36% by 120 days of age. Visible tumors were frequently found in the visceral organs of dead or diseased chickens by postmortem examination. For molecular diagnosis and viral isolation, feather pulps were collected from the chickens with suspected MD.

### 2.2. Viral Isolation and Identification

Feather pulps collected from the diseased chickens described above were used for viral isolation, as previously described [14]. Briefly, SPF duck or chicken embryos were obtained from the Harbin Veterinary Research Institute (HVRI), Chinese Academy of Agricultural Sciences (CAAS) for the preparation of duck embryo fibroblasts (DEFs) or chicken embryo fibroblasts (CEFs). Feather pulp-inoculated primary DEFs were incubated at 37 °C in an atmosphere containing 5% CO_2_, and blind passages were performed until cytopathogenic effects (CPEs) were observed. Viruses from infected cells with CPEs were plaque- purified in DEFs, and then the viruses were propagated and stored in liquid nitrogen.

Viruses were identified by a polymerase chain reaction (PCR)-based method targeting the *meq* gene and genomic 132-base pair repeat sequence (132bpr) of MDV, which we used to clearly distinguish between the vaccine and pathogenic wild-type MDV strains in a previous study [15]. DNA samples from inoculated CEFs were used as PCR templates, and the PCR primers used for MDV strain identification are listed in Table 1. Furthermore, an indirect immunofluorescence assay (IFA) was carried out to identify the viral plaques in CEFs using monoclonal antibodies, which were produced by our laboratory and are specific for the gI protein of MDV.

### 2.3. Screening for Causative Agents

Due to the multiple causes of oncosis in Chinese chicken flocks, it is necessary to detect and then cull chickens infected with one of several causative agents of oncogenicity. To detect avian leucosis virus (ALV), PCR and enzyme-linked immunosorbent assays (ELISAs) were performed, as previously described [16,17]. Additionally, PCR and IFA were used to detect reticuloendotheliosis virus (REV), as previously described [16,18]. Finally, a previously described PCR method was used to detect chicken infectious anemia virus (CIAV) [19].

### 2.4. Animal Experiments

The MDV strain BS/15 was used as a challenge virus, and the standard vv MDV strain Md5 was used as a reference strain. The CVI988 vaccine and 814 vaccine purchased from commercial companies were used in animal experiments to assess vaccine efficacy.

In total, 105 one-day-old SPF White Leghorn chickens were obtained from the Experimental Animal Center (EAC) of HVRI. The birds were housed in negative-pressure-filtered air isolators and were randomly divided into seven groups (*n* = 15 birds each). Vaccination was performed on day 1 and both vaccines were used at a dose of 2000 plaque forming units (PFU) in 200 μL dedicated diluent provided by the manufacturers, being administered via the subcutaneous route (groups 3 and 6: CVI988; groups 4 and 7: 814). In addition, groups 1, 2, 3, 5, and 6 were inoculated with 200 μL diluent in the same manner. On day 7 post-vaccination, MDV challenge was performed via the intra-abdominal route with 1000 PFU of MDV in 200-μL diluent (groups 2, 3, and 4: Md5; groups 5, 6, and 7: BS/15). Chickens of group 1 received the same amount of diluent in the same manner and served as controls.

In this study, the birds were observed daily for clinical signs of MD, and feather pulps were randomly plucked from five birds at 4, 7, 14, 21, 28, 35, 42, and 90 days post-challenge (dpc; *n* = 2 in the BS/15-challenged group, and *n* = 3 in the Md5-challenged control group at 90 dpc). In addition, the body weights and immune organ (bursa, thymus, and spleen) weights of the surviving chickens at the end-point of the experiment (90 dpc) were recorded for further analysis. The MD status of the experimental animals was estimated mainly by monitoring for early mortality syndrome, immune organ damage, and tumor formation, as described previously [14]. The days that various chickens died during the experimental period in each group were recorded for survival analysis.

The birds were randomly assigned to each group and numbered by workers at the EAC, HVRI. To ensure a blinded study, the group number was only known by the workers who dealt directly with the researchers. All animal experiments were approved by the ethical review board of HVRI, CAAS, and performed in accordance with approved animal care guidelines and protocols (approval number: SYXK (Heilongjiang) 2011022). These standard procedures included using as few animals as possible (*n* = 15 in each group); ensuring that the workers of the EAC of HVRI were trained strictly, in accordance with animal policy, and complied with all standard operating procedures; the feeding environment was clean, spacious, and comfortable, allowing the animals to move and feed freely; the poultry feed was nutritionally balanced, and the drinking water was fresh and clean; during the experimental operation, the treatments were applied using gentle movements, to avoid frightening the animals; and at the end of the experiment, the animals were euthanized immediately.

### 2.5. DNA Extraction and TaqMan Real-Time Polymerase Chain Teaction (qPCR)

The collected feather pulps were homogenized in phosphate buffer solution (PBS), and DNA was extracted using the AxyPrep Body Fluid Viral DNA/RNA Miniprep Kit (Corning Life Sciences Co., Ltd., Suzhou, China) according to the manufacturer’s instructions. The MDV meq gene and the chicken ovotransferrin gene were used as a real-time PCR (qPCR) target gene in the MDV genome and an internal reference gene in the host cell genome, respectively, and qPCR detection was performed as previously described [20,21].

### 2.6. Statistical Analysis

The vaccine protective index (PI) was calculated as previously described [22], using the following formula: PI = ((%MD in unvaccinated chickens; %MD in vaccinated chickens))/%MD in unvaccinated chickens) × 100. The absolute numbers of the MDV genome per million cells from the collected feather pulps were calculated based on the standard curves generated and were normalized to the viral load. The normalized viral load was calculated using the formula: normalized viral load = log_10_ ((MDV genome copy number/chicken genome copy number) × 10^6^). Viral load, body weight, immune organ index, and survival analysis data were analyzed using GraphPad Prism (Version 7.02; GraphPad Software, Inc., San Diego, CA, USA). Comparisons of the viral load between each group at each time point were determined using Multiple *t* tests (Holm–Sidak method, with alpha = 0.05, by GraphPad Prism), and the statistical significance of body weights and immune organ indexes between each group was evaluated using the same method. Survival patterns between each two groups were compared by Log-rank (Mantel–Cox) test. Differences were considered to be statistically significant at *p* < 0.05.

## 3. Results

### 3.1. Marek’s Disease Virus (MDV) Strain BS/15 Isolated in China

**Figure 1:**
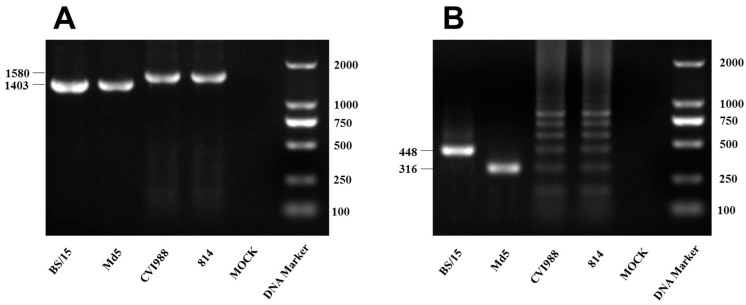
Detection of Marek’s disease virus (MDV) by polymerase chain reaction (PCR). DNA of MDV-infected chicken embryo fibroblasts (CEFs) was used as the template for PCR amplification. (**A**) PCR amplification of the *meq* gene of MDV. The PCR products of BS/15 and Md5 were 1403 base pair (bp) long, while those of CVI988 and 814 were 1580 bp, as they have a 177-bp insertion in the *meq* gene; (**B**) PCR amplification of the 132bpr of MDV. The PCR product of BS/15 132bpr was 448 bp long with a copy number of 3, while the PCR product length for Md5 was 316 bp with a copy number of 2, CVI988 and 814 showed PCR products 316–844 bp long and contained multiple copies of the 132bpr.

**Figure 2:**
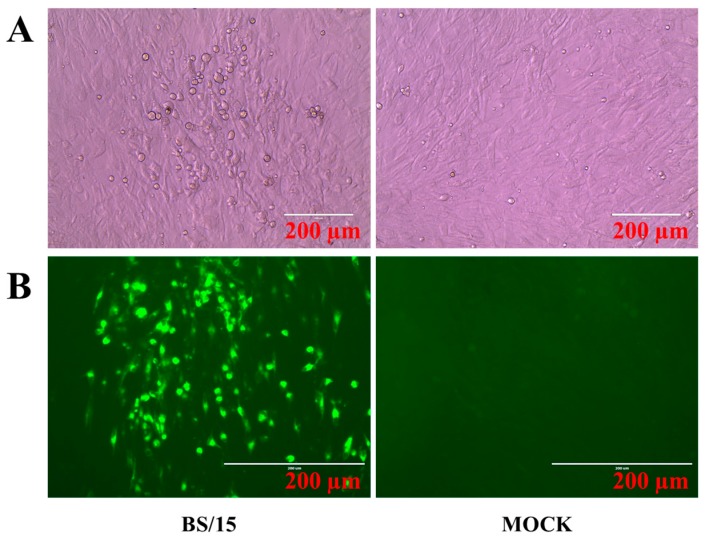
Viral plaques of CEF cultures caused by MDV infection, and immunofluorescence assays (IFAs). (**A**) Viral plaques of CEF cultures caused by MDV BS/15 were evident by light microscopy at 120 h post-inoculation; (**B**) Specific staining of the viral plaques with an MDV gI-specific monoclonal antibody was observed by fluorescence microscopy. Scale bar: 200 μm.

MDV strain BS/15 was isolated from diseased chickens from a layer farm located in the Jilin Province of China in 2015. PCR detection of MDV *meq* gene (Figure 1A) and 132bpr (Figure 1B) showed that MDV strain BS/15 has the unique molecular characteristics of pathogenic MDV strains. CEF cultures inoculated with BS/15 exhibited typical CPEs, consistent with those induced by MDV infection observed by light microscopy (Figure 2A). Then, the CPEs were confirmed in IFAs using MDV gI-specific monoclonal antibodies, and no CPEs were observed in uninfected CEF cultures using a fluorescence microscope (Figure 2B). In addition, no amplification products were obtained using PCR assays designed to detect ALV, REV, and CIAV, and the ALV ELISA test was negative, as was the IFA for REV; thus, the MDV isolate BS/15 was confirmed to be free of ALV, REV, and CIAV.

### 3.2. Virulence Studies of MDV BS/15 and Protective Potency Evaluation of Vaccines

#### 3.2.1. MD Incidence, Mortality, and Tumor Rates per Group

MD incidence, mortality, and tumor rates in each group were analyzed and are summarized in Table 2. No diseased chicken was observed in the control group and all of the unvaccinated BS/15- or Md5-challenged chickens developed MD during the experimental period. When vaccinated with vaccines CVI988 or 814, the MD incidences induced by BS/15 were 66.7% and 33.3%, respectively, while those of the vaccinated Md5-challenged groups were 7.1% and 0, respectively. Thus, the PIs of vaccines CVI988 and 814 against BS/15 were 33.3 and 66.7, respectively, while the PIs of vaccines CVI988 and 814 against Md5 were 92.9 and 100, respectively.

BS/15 and Md5 induced similar mortality in unvaccinated SPF chickens (85.7% and 80.0%, respectively). In addition, 53.3% and 33.3% of chickens died in the CVI988- or 814-vaccinated BS/15-challenged groups, respectively, while none of the vaccinated Md5-challenged chickens died. Furthermore, BS/15 infection caused many unvaccinated chickens (64.3%) to develop tumors in their visceral organs, whereas the tumor rate in Md5-challenged unvaccinated chickens was only 40.0%, showing that BS/15 has stronger oncogenicity. In contrast, only 13.3% and 20.0% of chickens in the CVI988- or 814-vaccinated, BS/15-challenged groups developed tumors, while vaccination completely prevented tumors induced by Md5.

#### 3.2.2. Survival Analysis

**Figure 3:**
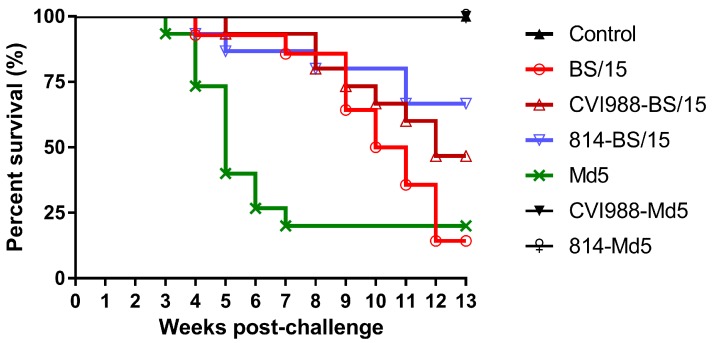
Survival curves for each group. The survival patterns between the BS/15-challenged group and Md5-challenged control group showed significant differences (*p* < 0.05) by Log-rank (Mantel-Cox) test.

The earliest deaths appeared at 22 dpc in the BS/15-challenged unvaccinated group, while the first death in the Md5-challenged reference group occurred at 19 dpc. Furthermore, deaths in unvaccinated BS/15-challenged chickens peaked at 9–12 weeks post-challenge (wpc), which was much later than that in the unvaccinated Md5-challenged group at 4–7 wpc. These results suggested that BS/15 infection induced death slower than Md5 infection in SPF chickens. Survival analysis revealed that the death patterns of chickens challenged with BS/15 and Md5 were different (*p* < 0.05), as shown in Figure 3.

#### 3.2.3. Developmental Disorders and Immune Organ Damages

**Figure 4:**
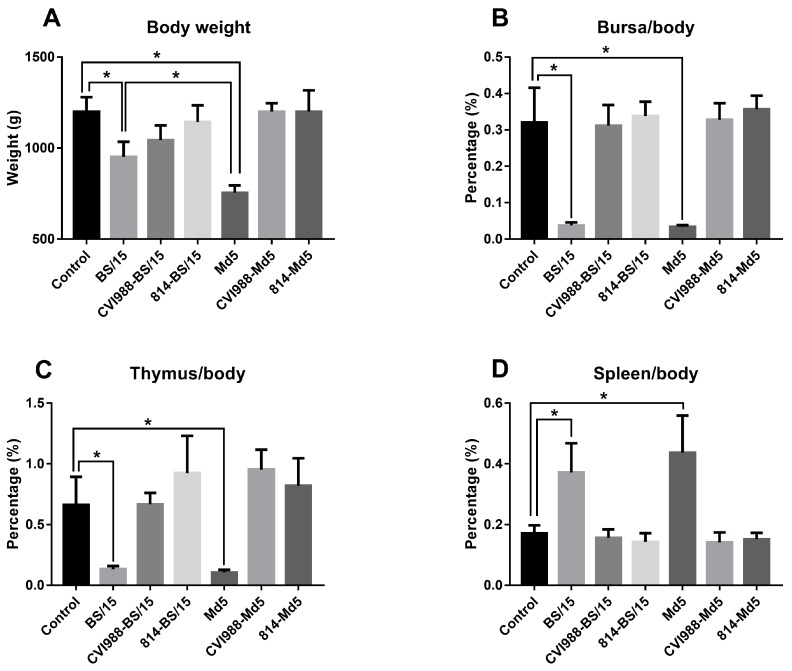
Body weights and ratios of immune organ weight/body weight in the surviving chickens of each group. The data are shown as mean with *SD* (*n* = 2 in the BS/15-challenged group, and *n* = 3 in the Md5-challenged control group, whereas *n* = 5 in the other groups), and differences were considered to be statistically significant at *p* < 0.05 (*). (**A**) Body weights of the surviving chickens in each group; (**B**) Ratios of bursa weight/body weight in the surviving chickens of each group; (**C**) Ratios of thymus weight/body weight in the surviving chickens of each group; (**D**) Ratios of spleen weight/body weight in the surviving chickens of each group.

At the end of the animal experiment at 90 dpc, the body weights and immune organ index of the surviving chickens in each group were calculated and analyzed (Figure 4). The body weights of BS/15- and Md5-challenged chickens (*n* = 2 and 3, respectively, whereas *n* = 5 in the other groups) were much lower than that of the control group (*p* < 0.05 for each comparison). Furthermore, the body weights of BS/15-challenged chickens were higher than those of Md5-challenged chickens (*p* < 0.05). These results suggested that both MDV strains affect the growth of chicken, and BS/15 inhibited chicken development less than Md5.

Immune organ damages are another typical symptom of MD. In this study, both BS/15 and Md5 infection could cause severe bursa and thymus atrophy (Figure 4B,C) and splenic enlargement (Figure 4D) compared to the controls (*p* < 0.05 for each comparison). Immune organ damages caused by BS/15 and Md5 showed no difference (*p* > 0.05 for each comparison).

Although the low survival number used for analysis in the BS/15- and Md5-challenged groups might allow for some statistical error, these data showed a trend towards the differential effects of MDV BS/15 and Md5 on chicken growth and immune suppression.

#### 3.2.4. MDV Genome Load in Feather Pulps

**Figure 5:**
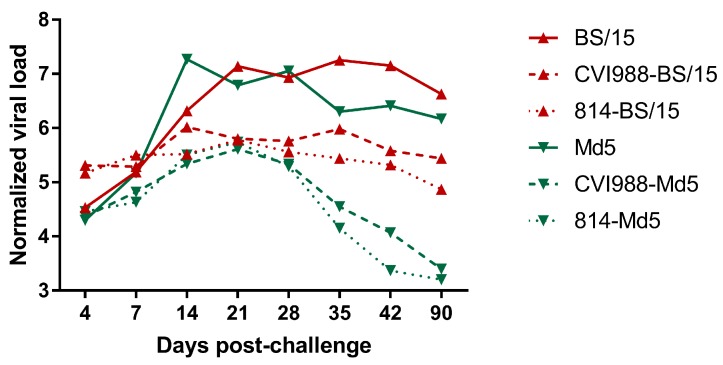
Normalized viral loads in the feather pulps of five birds from the various treatment groups at different time points (*n* = 2 in the BS/15-challenged group, and *n* = 3 in the Md5-challenged control group at 90 days post challenge; dpc). Normalized viral loads were calculated as the logarithm of the MDV copy numbers per million cells.

qPCR detection was performed with DNA from the feather pulps obtained from the BS/15- and Md5-challenged (*n* = 2 and 3, respectively, in the two groups at 90 dpc), vaccinated or unvaccinated chickens. The viral load of each sample was calculated and analyzed (Figure 5). The results showed that the MDV genome load of BS/15-challenged unvaccinated chickens was significantly lower than that of Md5-challenged unvaccinated chickens at 14 dpc, but became significantly higher than that of Md5-challenged unvaccinated chickens at 35 and 42 dpc (*p* < 0.05 for each comparison). In addition, the viral loads of BS/15-challenged vaccinated chickens were significantly higher than those of Md5-challenged vaccinated chickens between 35 and 90 dpc (*p* < 0.05 for each comparison). These results suggested that the vaccines could inhibit the replication of BS/15 and Md5 to some extent, and the vaccines better suppressed the replication of Md5 during the late period of viral infection.

Although the number of surviving animals in the BS/15- and Md5-challenged groups were less, which is likely to introduce an analytical error, this defect had little effect on the overall analysis of these results, especially before 42 dpc.

## 4. Discussion

Although MDV has been continually evolving and has obtained enhanced virulence over the last few decades, no report has described MDV stains with more virulence than vv+, and no trend of a large-scale MD outbreak has been shown. However, vaccination failure of MD has often occurred in China in recent years [14,23,24,25,26]. Here, a field MDV strain BS/15 was isolated from a Chinese chicken flock in 2015 that had been vaccinated with the commercial vaccine CVI988 and had developed severe MD. Determination of MDV pathotypes is useful for investigating the cause of excessive MD losses in vaccinated flocks [27]. The ADOL method of MDV pathotyping is widely recognized throughout the world. However, because of the limitations of ADOL (requirements for specific chicken types, large numbers of birds, and objective statistical methods to measure lesion responses), it is hardly used in other laboratories. Thus, researchers at the ADOL proposed that the pathotypes of MDV could be evaluated by comparing the pathogenicity and vaccine efficacy between the MDV isolates and the reference MDV strains in local SPF chickens [27]. In this study, to investigate the causes of vaccine immune failure, unvaccinated and CVI988- or 814-vaccinated SPF White Leghorn chickens were challenged with BS/15 and the standard vv MDV strain Md5 at day 7 post-vaccination, and the birds were observed for 90 dpc.

Animal experimental results showed that BS/15 and Md5 challenge both induced 100% MD incidence with similar mortality (85.7% and 80.0%, respectively) in unvaccinated SPF chickens. Unexpectedly, BS/15 induced much higher tumor rates than did Md5 in unvaccinated chickens (64.3% and 40.0%, respectively). In addition, although the number of surviving animals in the BS/15- and Md5-challenged groups at 90 dpc (*n* = 2 and 3, respectively) was less than that in the other groups (*n* = 5), which might allow for some statistical error, our data, to some extent, showed that BS/15 infection caused fewer serious effects on chicken growth and similar immune organ damages in unvaccinated chickens compared to Md5 (Figure 4). In vaccine protection tests, the vaccines CVI988 and 814 only provided PIs of 33.3 and 66.7 to SPF chickens against BS/15, while the PIs of vaccine CVI988 and 814 against Md5 were 92.9 and 100, respectively. Moreover, these results suggested that the virulence of BS/15 was similar to that of the vv MDV strain Md5 with different pathogenic characteristics, but vaccines could not provide effective protection against BS/15, especially the vaccine CVI988. These results confirmed that the virulence and vaccine resistance of MDV are different traits, as previously discussed [14,28]. Thus, we could not clearly classify the pathotype of MDV BS/15 by the ADOL pathotyping method. However, this did not affect our understanding of the pathogenicity and the potential threat of MDV BS/15. Data generated in this study demonstrated the existence of variant Chinese strains with known virulence with altered pathogenic characteristics, which were highly resistant to existing vaccines. Thus, the presence of such strains in the field may be a cause of MD vaccination failure in China.

The MDV genome load in the feather pulps of chickens plays an important role in monitoring the MD status and vaccination performance [21]. In this study, we quantified the viral load in chicken feather pulps (Figure 5). The results showed that the viral loads of BS/15-challenged unvaccinated chickens were lower than those of Md5-challenged unvaccinated chickens at 14 dpc (*p* < 0.05), but were higher than those of Md5-challenged unvaccinated chickens at 35 and 42 dpc (*p* < 0.05 for each comparison). The lower viral loads observed in BS/15-challenged unvaccinated chickens during an early experimental period (about 7–21 dpc) may be associated with the delayed pathogenesis of BS/15. As shown in Figure 3, increasing deaths occurred in the BS/15-challenged group beginning at 9 wpc, while none of the chickens died in the Md5-challenged control group after 7 wpc. These data indicated that chickens in the BS/15-challenged group were suffering from MD, while some chickens in the Md5-challenged control group were already resistant to MD at 35 and 42 dpc, as consistently revealed by measuring the viral load in the two groups at the same time point. Furthermore, the vaccines could not effectively inhibit the replication of BS/15 compared to Md5 after 28 dpc (*p* < 0.05 for each comparison), which may explain the different protective effects of vaccines against BS/15 and Md5 to a certain extent.

Previous epidemiological studies have demonstrated the lack of efficacy of vaccines against Chinese MDV isolates with virulence of no obvious enhancement. SD2012-1 has a long latent period and causes subsequent death with a mortality rate of 70.3% in unvaccinated SPF chickens; however, SD2012-1 can break through the protection provided by vaccine HVT (FC126 strain) or bivalent vaccine HVT plus SB-1, causing mortality rates of over 60% in vaccinated SPF chickens [23]. In another study, the CVI988 vaccine could only protect 83.0% of SPF chickens from SX1301 infection, despite the low mortality rate of 57.0% caused by SX1301 in unvaccinated SPF chickens [24]. In our previous study, the field MDV strain LTS induced a very low mortality (23.1% by 60 dpc) in unvaccinated chickens, but the vaccine CVI988 could not provide efficient protection (PI: 66.7) against LTS [14]. In addition, MDV isolates ZY/1203 and WC/1110 induced very low MD mortality in unvaccinated chickens (20.5% and 21.1% by 60 dpc, respectively) and were similar to that of the standard v MDV strain GA (23.5% by 60 dpc). Although vaccine CVI988 could provide good protection against ZY/1203 and WC/1110 (PI as 82.4 and 83.3, respectively), vaccine CVI988 could not provide complete protection against them compared to that of vaccine CVI988 against GA (PI: 100) [25].

These studies suggested that the virulence of field MDV strains prevalent in China may not have increased in recent years, but several newly isolated MDV strains had obtained the ability to counter the vaccines, such as strain LTS isolated previously and strain BS/15 isolated in this study. We further inferred that the evolution patterns of MDV strains prevalent in China might have changed and no longer synchronously enhanced virulence and vaccine resistance. Thus, the newly isolated Chinese MDV strains often did not show stronger pathogenicity, but their survivability in vaccinated hosts was stronger, leading to increased vaccination failure in recent years. Therefore, the emergence of variant strains and their potential harm to the poultry industry need to be constantly monitored. Given the delayed pathogenesis of these variant MDV strains, their popularity in the field may be a greater threat to laying and breeding chickens than to meat-producing chickens. Thus, we are considering the necessity and feasibility of classifying these variant MDV strains as a new pathotype (such as “late virulence”), to facilitate the analysis and study of these variant strains in the future.

## 5. Conclusions

To conclude, a field MDV strain BS/15 was isolated from vaccinated chicken flocks in China. Animal experiments showed that the virulence of BS/15 was similar to the reference vv MDV strain Md5 with different pathogenic characteristics. The commercial vaccines could not provide effective protection against BS/15; BS/15, in particular, BS/15 could badly counter the CVI988 vaccine (PI: 33.3). Our study suggested the existence of variant MDV strains, which did not have particularly strong virulence but showed that powerful vaccine resistance exists in China, and these MDV strains may be the etiological cause of MD vaccination failure. Thus, improved vaccines are needed for MDV control. Furthermore, our study provided evidence that the MDV evolution does not always synchronously enhance virulence and vaccine resistance.

## Figures and Tables

**Table 1:** Polymerase chain reaction (PCR) primers for Marek’s disease virus (MDV) strain identification.

Target	Primer Sequence	Product Size (bp)
*meq*	F: 5′-GGGAAATGACAGGTGAATTGTG-3′	1403/1580 ^a^
	R: 5′-TAAGGAAAATTTGTTACCCCAG-3′	
*132bpr*	F: 5′-TGCGATGAAAGTGCTATGGAGG-3′	316–844 ^b^
	R: 5′-GAGAATCCCTATGAGAAAGCGC-3′	

^a^ Exact size is strain-dependent, based on inclusion of a 177-bp insertion; ^b^ Exact size depends on 132-base pair repeat sequence (132bpr) copy number.

**Table 2:** Marek’s disease (MD) incidence, mortality, and tumor rates in each group at 90 dpc.

Vaccine	Challenge	MD Incidence *Diseased*/*Total* (%)	PI	Mortality *Deaths*/*Total* (%)	Tumor Incidence ^a^
None	None	0/14 (0%)	-	0/14 (0%)	0%
None	Md5	15/15 (100%)	-	12/15 (80.0%)	40.0%
CVI988	Md5	1/14 (7.1%)	92.9	0/14 (0%)	0%
814	Md5	0/13 (0%)	100	0/14 (0%)	0%
None	BS/15	14/14 (100%)	-	12/14 (85.7%)	64.3%
CVI988	BS/15	10/15 (66.7%)	33.3	8/15 (53.3%)	13.3%
814	BS/15	5/15 (33.3%)	66.7	5/15 (33.3%)	20.0%

PI, protective index; ^a^ Percent of birds that developed tumors; dpc, days post-challenge.
